# Parameters impacting the live birth rate per transfer after frozen single euploid blastocyst transfer

**DOI:** 10.1371/journal.pone.0227619

**Published:** 2020-01-13

**Authors:** Fazilet Kubra Boynukalin, Meral Gultomruk, Sabri Cavkaytar, Emre Turgut, Necati Findikli, Munevver Serdarogullari, Onder Coban, Zalihe Yarkiner, Carmen Rubio, Mustafa Bahceci

**Affiliations:** 1 Department of Reproductive Endocrinology and IVF Center, Bahceci Health Group, Istanbul, Turkey; 2 Department of Embryology and R&D Center, Bahceci Health Group, Center, Istanbul, Turkey; 3 Cyprus Science University, Kyrenia, Cyprus; 4 Igenomix, Valencia, Spain; IRCCS San Raffaele Scientific Institute, ITALY

## Abstract

**Background:**

To assess the predictive value of patient characteristics, controlled ovarian stimulation and embryological parameters on the live birth outcome of single euploid frozen-warmed blastocyst transfer (FBT).

**Methods:**

This was a retrospective cohort study including 707 single FBTs after preimplantation genetic testing for aneuploidy (PGT-A) that were performed from October 1, 2015, to January 1, 2018. The effects of patient-, cycle- and embryology-related parameters on the live birth outcome after FBT were assessed.

**Results:**

In the subgroup analysis based on live birth, patients who achieved a live birth had a significantly lower body mass index (BMI) than patients who did not achieve a live birth (22.7 (21.5–24.6) kg/m^2^ vs 27 (24–29.2) kg/m^2^, p<0.001). The percentage of blastocysts with inner cell mass (ICM) A or B was significantly higher among patients achieving a live birth, at 91.6% vs. 82.6% (p<0.001). Day-5 biopsies were also more prevalent among patients achieving a live birth, at 82.9% vs 68.1% (p<0.001). On the other hand, the mitochondrial DNA (mtDNA) levels were significantly lower among cases with a successful live birth, at 18.7 (15.45–23.68) vs 20.55 (16.43–25.22) (p = 0.001). The logistic regression analysis showed that BMI (p<0.001, OR: 0.789, 95% CI [0.734–0.848]), day of trophectoderm (TE) biopsy (p<0.001, OR: 0.336, 95% CI [0.189–0.598]) and number of previous miscarriages (p = 0.004, OR: 0.733, 95% CI [0.594–0.906]) were significantly correlated with live birth. Patients with elevated BMIs, cycles in which embryos were biopsied on day-6 and a higher number of miscarriages were at increased risks of reduced live birth rates.

**Conclusion:**

A high BMI, an embryo biopsy on day-6 and a high number of miscarriages negatively affect the live birth rate after single euploid FBT.

## Introduction

Chromosomal aneuploidy is present in approximately 50% of embryos throughout preimplantation development and is a consequence of errors occurring during gametogenesis and early mitotic divisions that lead to implantation failure, spontaneous abortion, and the birth of a child with a trisomic condition [1]. The main goal of preimplantation genetic testing for aneuploidy (PGT-A) is to select euploid embryos for subsequent transfer. PGT-A has been performed in in-vitro fertilization (IVF)/intracytoplasmic sperm injection (ICSI) for different indications, such as advanced maternal age (AMA), repeated implantation failure (RIF), recurrent miscarriage (RM), severe male factor infertility and elective single-embryo transfer (eSET) [2].

After day 3, PGT-A of nucleated blastomeres by fluorescence in situ hybridization (FISH) failed to demonstrate an improvement in clinical outcomes [3–10]. The emergence of newer technologies, such as array comparative genomic hybridization (aCGH), single nucleotide polymorphism (SNP) array, quantitative polymerase chain reaction (qPCR) and next-generation sequencing (NGS) with multicellular trophectoderm biopsy have led to more favorable outcomes with comprehensive chromosomal screening [11–14]. However, not all IVF laboratories utilizing PGT-A have demonstrated improved outcomes with this approach. The inconsistencies in the results obtained from laboratories utilizing PGT-A are due to the fact that PGT-A is a technology that relies heavily on multiple laboratory procedures. Extended embryo culture, trophectoderm biopsy and cryopreservation with vitrification are all essential components that are required to obtain optimal results by PGT-A. In addition to embryological parameters, clinical variables, such as parameters for controlled ovarian stimulation (COH) and those for endometrial preparation for frozen embryo transfer (FET) are associated with differences in IVF/ICSI outcomes, adding to the complexity of achieving IVF/ICSI success. Nevertheless, current data are very limited and rely mostly on patients with a good prognostic background.

The predictive factors for live birth after IVF/ICSI treatment with eSET have long been studied. In a recent prospective observational cohort study, eSETs in 8,451 IVF/ICSI treatments in 5,699 unselected consecutive couples were analyzed, and embryo score, treatment history, number of oocytes, total dose of FSH administered, female age, infertility cause, endometrial thickness, and female height were all found to be independent predictors of live birth [15]. However, there is a paucity of data on the predictive factors for live birth after single euploid frozen-warmed blastocyst transfer (FBT).

In this retrospective analysis, our aim was to determine which factors were associated with live birth rates after single euploid FBT.

## Material and methods

**Fig 1:**
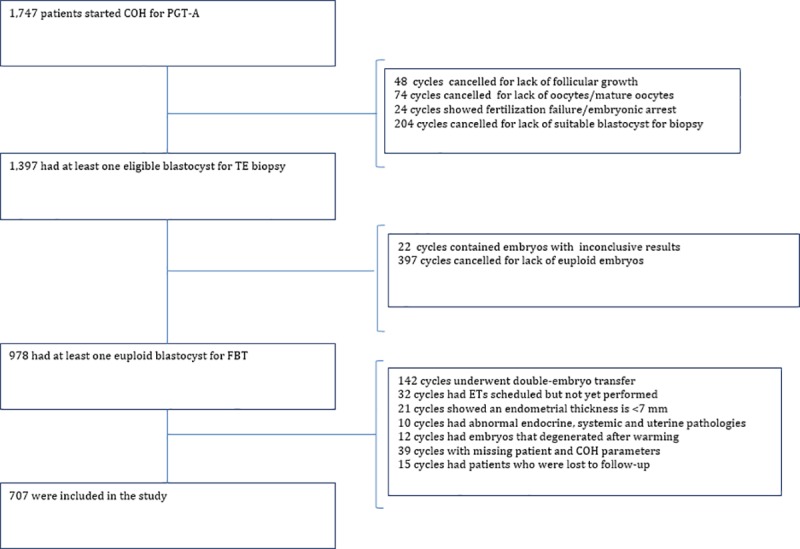
Flowchart of cycles included in the study.

Data from 1,747 cycles with intent for PGT-A were collected from Bahceci Fulya IVF Center (Istanbul) from October 1, 2015, to January 1, 2018. Of these cycles, 1,397 reached the embryo biopsy stage and 978 were found to have at least one euploid embryo for FBT. The study population consisted of cycles in which women were 20–45 years of age, undergoing ICSI-PGT and employing TE biopsy using 24-chromosome NGS. The exclusion criteria were endocrine or systemic pathologies, uterine anomalies or pathologies, unilateral or bilateral hydrosalpinx, and karyotypic abnormalities (either maternal or paternal). Only 707 single euploid FBT cycles were found to be eligible for inclusion in the study, as depicted in Fig 1. PGT-A indications were as follows: RIF (392/707, 55.4%), AMA (120/707, 17%), RM (69/707, 9.8%) and multiple indications (126/707, 17.8%). As recommended by the American Society of Reproductive Medicine (ASRM), women with RM had a complete RM workup that included blood work for parental karyotypes and to detect the presence of antiphospholipid antibodies, including anti-cardiolipin antibody, lupus anticoagulant and beta-2-glycoprotein, as well as a uterine cavity evaluation. Women were also routinely screened for hypothyroidism and hyperprolactinemia by measuring the levels of serum thyroid-stimulating hormone and prolactin. Patients with unknown etiology for RM were included in this study.

All patients provided informed consent for analysis of their identified data before undergoing IVF procedures. The study protocol was approved by the Bilim University Ethics Committee (4414529/2015-61).

### Ovarian stimulation, oocyte retrieval, denudation, ICSI and embryo culture

COH was performed with the GnRH antagonist protocol. Recombinant FSH (150–300 IU, Gonal-F; Serono) and/or hMG (75–150) IU; (Merional; IBSA) was administered on day 2 of the menstrual period. Starting on the sixth day of controlled ovarian stimulation, the ovarian response was monitored by serial transvaginal ultrasound (TV-USG) and by measuring serum E_2_ and P_4_ levels. When the leading follicle exceeded 13 mm in diameter, 0.25 mg of GnRH antagonist (Cetrotide; Serono) was started daily until the day of the last trigger. When at least two follicles reached 18 mm in diameter, patients were administered 250 μg of human chorionic gonadotropin (hCG; Ovitrelle, Serono) or 0.2 mg of triptorelin (Gonapeptyl, Ferring), and oocyte retrieval was scheduled 35 hours after the trigger administration [16].

The oocyte retrieval, denudation, and ICSI procedures were performed as described previously by Serdarogullari et al. [17]. ICSI was the fertilization method in all of the cycles included in this study. After microinjection, oocytes were cultured individually in a special pre-equilibrated culture dish. In our study, single-step media, namely, Continuous Single Culture Complete (CSCM-C) with Human Serum Albumin (Irvine Scientific) was used for embryo culture throughout the culture period.

### Embryo morphology assessment and trophectoderm biopsy

Embryo culture was performed in benchtop incubators (MIRI, ESCO Medical, Singapore) until day-6 of embryo development with daily morphological grading by Gardner and Schoolcraft [18]. The developmental characteristics of each individual embryo were recorded. Blastocyst morphology evaluations were performed at 114 hours (day-5) and 138 hours (day-6) after ICSI. Assisted hatching (AH) was applied to each embryo with a hole of approximately 20 μm using a laser pulse (OCTAX Navilase) on day 3 of embryo development. After day-3 laser application, embryos were transferred into new fresh medium (CSCM-C with Human Serum Albumin) until the day of the biopsy. Biopsy of each embryo was performed in 5-μL droplets of mHTF with Gentamicin (mHTF, Irvine Scientific, CA, USA) containing 10% SSS (Irvine Scientific, CA, USA). On day-5 of embryo development, AH-applied blastocysts that displayed a herniating trophectoderm during embryo scoring underwent biopsy. The remaining blastocysts that did not display herniation were further cultured until day-6 of embryo development. When available, a second embryo evaluation was performed 4–6 hours after the routine embryo grading schedule. Only blastocysts with herniated trophectoderm cells underwent biopsy on days 5 and 6. Trophectoderm biopsy was performed using the pulling method, as previously described by Zhao et al. [19]. Approximately five to eight cells were removed from the trophectoderm, and extracted cells were placed in polymerase chain reaction tubes and kept frozen at -20°C until PGT-A.

### Embryo vitrification and warming procedures

Embryo vitrification and warming procedures were achieved using a commercial vitrification kit (Vit Kit ^®^-Freeze, 90133-SO, Irvine Scientific) and vitrification warming kit (Vit Kit ^®^-Thaw, 90137-SO, Irvine Scientific). In all cases, an open carrier device (Cryotech, Reprolife, Japan) was used, and embryos were vitrified within 2 hours after trophectoderm biopsy (with a mean of 1 hour and 20 minutes) Vitrification and warming were performed as previously described [17].

### Evaluation of viability after warming and extended culture

After the warming procedure was completed, the embryo was transferred to an equilibrated culture dish up to embryo transfer. Blastocyst grading was performed 2–3 hours after the warming procedure according to the classification of Gardner and Scoolcraft. Viability after warming was quantified and classified according to the percentage of survived (intact) blastomeres (100%, ≥50%, <50%, 0%) that were present in a blastocyst stage embryo and the blastocoel re-expansion ability. Only embryos that survived with fully intact morphology (100%) comprising a distinguishable inner cell mass and trophoblast were included in the study.

### Endometrial preparation and support

Endometrial preparation for ET involved hormone replacement therapy. Briefly, each woman was administered oral estrogen (Estrofem, Novo Nordisk, Istanbul, Turkey) according to an incremental regime: 4 mg/day on days 1–4, 6 mg/day on days 5–8, and 8 mg/day on days 9–12. TV-USG was performed on day 13 to measure endometrial thickness, and if endometrial thickness was <7 mm, the cycle was cancelled. The serum P_4_ concentration was also measured; if this concentration was >1.5 ng/ml, the cycle was also cancelled. Estrogen supplementation was continued at 8 mg/day, and intramuscular (IM) progesterone (Progestan, Koçak Farma, Turkey) supplementation at 50 mg/day was started as previously described [20]. The embryo transfer was performed on the 6th day of progesterone administration. Oral estrogen was continued until the 7th week of pregnancy, and IM progesterone was continued until the 10th week of pregnancy.

### Preimplantation genetic test for aneuploidy (PGT-A) and mitochondrial DNA (mtDNA) analysis

The NGS platform (Reproseq PGS Kit, Life Technologies/Thermo Fisher, USA) used in this study has previously been validated and published elsewhere [21, 22]. Whole-genome amplification (WGA) and DNA barcoding were performed using the Ion ReproSeq PGS kit (Thermo Fisher Scientific, MA, USA). Automated template preparation and chip loading were automated with IonChef ^TM^ (Thermo Fisher Scientific). Sequencing steps were subsequently performed in a PGM sequencing machine using a 318 chip or in a S5 TM XL sequencer (Thermo Fisher Scientific) using a 530 chip. Data analysis was performed using version 5.4 of the Ion Reporter software (Thermo Fisher Scientific). Embryos were diagnosed as euploid, aneuploid or chaotic abnormal.

For the calculations of the mtDNA ratios, an optimized algorithm was applied that used the output dataset obtained from the NGS analysis, which comprised a mixture of mtDNA reads and nuclear DNA (nDNA) reads. To calculate the relative mtDNA copy number score in embryos, the numbers of reads after filtering were mapped to the mitochondrial genome and were divided by the number of reads that mapped to the nuclear genome as previously described and called mitoscore [23].

### Pregnancy outcome measurements

The serum human chorionic gonadotrophin (β-hCG) level was measured 12 days after embryo transfer and regarded as positive if it was more than 5 IU. The clinical pregnancy rate per embryo transfer was determined by dividing the number of embryo transfers having a gestational sac observed by means of ultrasound over the number of embryo transfers. The live birth rate per embryo transfer was defined as the number of deliveries divided by the number of embryo transfers. The miscarriage rate was calculated by dividing the number of pregnancies with a gestational sac that could not reach delivery by the total number of pregnancies with a gestational sac.

### Statistical analysis

All statistical analyses were performed with SPSS for Windows software package version 20 (SPSS, Chicago, USA). A p-value of p≤0.05 was considered to indicate statistical significance for all statistical tests. Continuous quantitative variables investigated in the study were reported as the median (quartile 1-quartile 3).

A chi-square test was used to analyze the proportions of β-hCG positivity, clinical pregnancy, live birth and miscarriage rates in maternal age groups.

Distributions of continuous parameters were tested with the Kolmogorov-Smirnov test to determine whether each variable followed a normal distribution. 707 patients were then divided into two groups of outcomes of live birth; whether a patient has had a live birth (n = 403) or not (n = 304). Since the continuous variables were not found to follow a normal distribution, a nonparametric equivalent test, namely, the independent-samples median test, was used to compare the medians of two groups of live birth outcomes with respect to the patient characteristics and cycle parameters. Differences in euploid hatching embryo characteristics in ICM score, trophectoderm score and day of embryo biopsied groups are compared between patients who have and haven’t had a live birth by using chi-square test. At the same time, the differences in the median scores of mitoscore among patients who had and didn’t have a live birth are compared by using nonparametric independent samples median test.

Finally, a binary logistic regression model was performed to evaluate the factors affecting the live birth outcome. In performing the binary logistic regression analysis, the forward stepwise procedure and likelihood ratio statistics were used as the criteria for removing the variables from the model. Maternal age, paternal age, BMI, infertility diagnose, number of previous attempts, number of previous miscarriages, duration of stimulation, total gonadotropin dosage used, E_2_ and P_4_ levels on trigger day, endometrial thickness, E_2_ and P_4_ levels 6 days before the FBT cycle, mitoscore, ICM score, trophectoderm score and day of embryo biopsy were used as factors and covariates in the binary logistic regression model. Odds ratios and corresponding 95% confidence intervals are reported for each significant variable to analyze the effect of each parameter.

## Results

During the study period, a total of 707 single euploid FBT cycles employing NGS were included. Overall, the clinical outcomes in this study were as follows: positive β-hCG rate: 71% (502/707); clinical pregnancy rate: 67.2% (475/707); live birth rate: 57% (403/707); and miscarriage rate: 15.2% (72/475). Table 1 summarizes the pregnancy outcomes per euploid ET across all maternal age groups. Of note, the outcome rates per transfer for cycles with the use of NGS-based PGT-A remained relatively constant across all maternal age groups.

**Table 1:** Pregnancy outcomes for single euploid embryo transfers stratified by maternal age.

Age	β-hCG Positivity (%)	Clinical Pregnancy (%)	Live Birth (%)	Miscarriage (%)
**All**	502/707 (71)	475/707 (67.2)	403/707 (57)	72/475 (15.2)
**< 35**	190/259 (73.4)	174/259 (67.2)	158/259 (61.0)	16/174 (9.2)
**35–37**	128/195 (65.6)	124/195 (63.6)	101/195 (51.8)	23/195 (11.8)
**38–40**	100/136 (73.5)	97/136 (71.3)	77/136 (56.6)	20/136 (14.7)
**41–42**	64/88 (72.7)	60/88 (68.2)	49/88 (55.7)	11/88 (12.5)
**>42**	20/29 (60.0)	20/29 (60.0)	18/29 (62.1)	2/29 (6.9)

Chi-square p-value for **β hCG Positivity (%)** = 0.402

Chi-square p-value for **Clinical Pregnancy (%)** = 0.684

Chi-square p-value for **Live Birth (%)** = 0.198

Chi-square p-value for **Miscarriage (%)** = 0.282

A total of 707 single euploid FBTs were grouped based on the live birth outcome. Group I included cycles that ended in live birth (n = 403), whereas Group II consisted of cycles that did not (n = 304). Regarding patient characteristics (female age, type of infertility, number of previous attempts, number of previous miscarriages, infertility diagnose), there were no significant differences between the two groups. BMI was found to be significantly higher in Group II than in Group I (27 (24–29.2) kg/m^2^ vs 22.7 (21.5–24.6) kg/m^2^, p<0.001). In all cycles, sperm count, controlled ovarian stimulation parameters, number of oocytes retrieved, number of mature oocytes (MII), and number and rate of fertilized oocytes with two pronuclei (2PN) were not significantly different between groups. Trigger day E_2_ level was significantly higher in Group I than in Group II (1428 (713–2237) pg/mL vs (1050 (658–2129) pg/mL, p = 0.003). Regarding FBT cycle parameters, neither endometrial thickness nor E_2_ or P_4_ levels on the day of progesterone initiation were significantly different between the two groups (Table 2).

**Table 2:** Patient characteristics, ovarian stimulation variables and variables during the FBT cycles in comparison with patients with or without live birth.

	Live birth (+) (n = 403)	Live birth (-) (n = 304)	p
**Maternal age (years)**	35 (32–39)	36 (33–39)	0.503
**Paternal age (years)**	37 (30–43)	37 (30–42)	0.528
**BMI (kg/m2)**	22.70 (21.50–24.60)	27 (24–29.2)	<0.001
**Type of infertility**			
**Primary infertility**	358/403 (88.8)	269/304 (88.5)	0.885
**Secondary infertility**	45/403 (11.2)	35/304 (11.5)	
**Infertility diagnosis**			
**Tubal**	40/403 (9.9)	31/304 (10.2)	0.185
**DOR**	65/403 (16.1)	58/304 (19.1)	
**Endometriosis**	44/403 (10.9)	30/304 (9.9)	
**Male factor**	102/403 (25.3)	81/304 (26.6)	
**Multiple indication**	35/403 (8.7)	32/304 (10.5)	
**PCOS**	48/403 (11.9)	42/304 (13.8)	
**Unexplained**	69/403 (17.1)	30/304 (9.9)	
**Number of previous attempts**	2 (1–4)	3 (2–4)	0.953
**Number of previous miscarriages**	0 (0–1)	1 (0–2)	0.248
**Duration of stimulation (days)**	9 (8–10)	9 (8–10)	0.101
**Total gonadotropin dosage used (IU)**	2250 (1650–2850)	2235 (1662.50–3000)	0.926
**Sperm Count (million/mL)**	35 (12–50)	32 (13.75–52)	0.399
**Number of oocytes retrieved**	11 (7–16)	11 (6–16.50)	0.698
**Number of M2 oocytes**	9 (5–12)	9 (4–13)	0.877
**Number of 2PN**	7 (4–10)	7 (3–10)	0.902
**Fertilization Rate (%)**	80 (67.33–93.73)	80 (72.47–100)	0.732
**Endometrial thickness (mm) on trigger day**	9 (8.10–10)	9 (8.10–10)	0.792
**Hormone levels (on trigger day)**			
**E**_**2**_ **(pg/mL)**	1428 (713–2237)	1050 (658–2129)	0.003
**P**_**4**_ **(ng/mL)**	0.62 (0.31–0.88)	0.66 (0.32–1.10)	0.256
**Hormone levels** **(6 days before FBT cycle)**			
**E**_**2**_ **(pg/mL)**	305 (233–405)	319 (232–442.50)	0.593
**P**_**4**_ **(ng/mL)**	0.15 (0.08–0.25)	0.13 (0.085–0.25)	0.205
**Endometrial thickness (mm)**	9 (8.10–10)	9 (8.10–10)	0.792

Values are presented as the median (quartile 1-quartile 3) or number (percentage).

DOR, PCOS, BMI, M2, 2PN and hCG denote diminished ovarian reserve, polycystic ovary syndrome, body mass index, mature oocyte, 2 pro-nuclei and human chorionic gonadotropin, respectively.

P values were calculated by means of the chi-square test, and independent samples median test.

The embryo development characteristics are presented in Table 3. The percentage of ICM A or B was found to be significantly higher in patients with a live birth than in those without a live birth (91.6% vs. 82.6%, p<0.001). Day-5 biopsied embryos were also more prevalent in Group I than Group II; 82.9% vs. 68.1% (p<0.001). On the other hand, the mitoscore levels were lower for cases having a successful live birth, at 18.7 (15.45–23.68) vs 20.55 (16.43–25.22) (p = 0.001).

**Table 3:** Euploid hatching embryo characteristics of patients with or without a live birth.

Embryo Characteristics	Live birth (+) (n = 403)	Live birth (-) (n = 304)	p
**ICM score (n, %)**			<0.001
**A or B**	369/394 (91.6)	251/296 (82.6)	
**C**	25/394 (6.2)	45/296 (14.8)	
**Trophectoderm score (n, %)**			0.061
**A or B**	172/394 (42.7)	111/296 (36.5)	
**C**	222/394 (55.1)	185/296 (60.9)	
**Mitoscore**	18.7 (15.45–23.68)	20.55 (16.43–25.22)	0.001
**Day of embryo biopsy (n, %)**			<0.001
**5**	334/403 (82.9)	207/304 (68.1)	
**6**	69/403 (17.1)	97/304 (31.9)	

Values are presented as the number (percentage) or median (quartile 1-quartile 3).

ICM denotes inner cell mass.

p values were calculated by means of the chi-square test, and independent samples median test.

In the present study, patient characteristics, ovarian stimulation variables, embryo development characteristics and variables associated with FBT cycles were also used to identify possible factors that could impact the live birth outcome. When all the parameters were assessed together to identify which covariates and factors affected the live birth outcome, BMI, number of previous miscarriages and day of TE biopsy were found to be significant. To evaluate the level of the effect of each of these parameters on the live birth outcome, the odds ratio was used (Table 4). The negative beta value indicated that when the day of the blastocyst TE biopsy was changed from day-5 to day-6, the probability of having a live birth decreased (OR: 0.336, 95% CI 0.189–0.598, p<0.001). In addition, as the number of miscarriage increased, the live birth rate decreased (Table 5). Thus, an increase in the number of miscarriages per unit decreases the probability of having a live birth (OR: 0.733, 95% CI 0.594–0.906, p = 0.004). When the BMI of a patient was taken into consideration, the results of the binary logistic regression analysis showed that an increase of one unit in the BMI value decreased the probability of having a live birth by 0.211 (OR: 0.789, 95% CI 0.734–0.848, p<0.001) (Table 4). Neither the parameters for controlled ovarian stimulation nor the regimen for endometrial preparation during FBT, such as the levels of E_2_ and P_4_, number of COCs, number of M2 oocytes, fertilization rate or endometrial thickness, showed any effects on the live birth rate.

**Table 4:** Binary Logistic Regression Model using Forward Stepwise Model (Likelihood Ratio).

Variable	B	S.E.	Wald	df	Sig.	OR	95% CI for OR	
							Lower	Upper
**BMI**	-0.237	0.037	41.10	1	<0.001	0.789	0.734	0.848
**Day-6**	-1.089	0.293	13.817	1	<0.001	0.336	0.189	0.598
**Day-5**	/	/	/	/	/	/	/	/
**Number of previous miscarriages**	-0.310	0.108	8.298	1	0.004	0.733	0.594	0.906
**Constant**	7.088	0.949	55.772	1	<0.001	1197.690		

Nagelkerke R-square = 0.266

**Table 5:** Number of miscarriages and live births.

		Live Births		Total
		No	Yes	
**Number of previous miscarriages**	**0**	151 (40.8%)	219 (59.2%)	370
	**1**	68 (40.2%)	101 (59.8%)	169
	**2**	55 (48.7%)	58 (51.3%)	113
	**3**	17 (50.0%)	17 (50.0%)	34
	**4**	6 (54.5%)	5 (45.5%)	11
	**5**	7 (70.0%)	3 (30.0%)	10
**Total**		304	403	707

## Discussion

This study aimed to evaluate and determine which factors affect live birth outcomes after single euploid FBT cycles. Our findings indicate that BMI, day of TE biopsy and number of previous miscarriages are significantly associated with the live birth rate per transfer.

Increasing maternal age is related to decreasing success in both spontaneous and IVF/ICSI-mediated conception [24]. Maternal age is among the strongest predictors of the success of IVF/ICSI treatments [25]. The aneuploidy rates in both oocytes and in vitro-produced embryos increase with increasing female age [26, 27]. PGT-A allows patients to avoid the transfer of aneuploid blastocysts, which is related to implantation failure, miscarriage and the birth of an affected child. Regarding the patient-specific variables, no impact of maternal age on live birth after a single euploid FBT was observed in this study. It is important to note that once a euploid embryo is identified, the implantation potential and miscarriage risk are not related to maternal age. Consistent with the outcome observed in the BEST trial, which employed single euploid ETs, the outcome in this study is independent of age, and eSET can be considered a feasible strategy that has satisfactory live birth rates and dramatically reduces the risk of multiple pregnancy [11]. However, it should also be noted that obtaining one euploid blastocyst becomes more challenging with increasing maternal age since there are numerous adverse factors that are associated with cycle cancellation, such as the lack of follicular development, unsuccessful oocyte retrieval and decreasing blastulation rate. In addition, the lack of an association with maternal age seems to be relevant for the transfer but not for the oocyte retrieval.

Data on the potential effects of elevated BMI on fertility treatment outcomes are conflicting. Multiple studies have reported no significant adverse effects of elevated BMI on IVF outcomes [28–32]; however, others have found associations between elevated BMI and higher gonadotropin requirements, fewer oocytes collected, higher cancellation rates, reduced pregnancy and live birth rates, and higher miscarriage rates [33–45]. Whether obesity has a negative effect on implantation and postimplantation events and on oocyte and embryo quality remains uncertain. Although euploidy rate and BMI were not found to be related in a recent retrospective analysis [46], other recent studies have shown that the chances of a live birth after euploid embryo transfers were significantly reduced in overweight women, especially in those with a BMI in the obese range (BMI ≥30) [47]. Supporting these data, a product of conception (POC) analysis showed the overpresentation of euploidy in obese patients compared with lean patients [48], indicating that endometrial changes associated with obesity, such as endometrial morphology, steroid receptors or leukocyte populations, may have a detrimental effect on endometrial receptivity [37]. In our current study, multivariate logistic regression analysis showed that BMI was an independent factor for live birth, which suggests a potential problem for endometrial receptivity.

According to the logistic regression analysis, TE score and ICM score were not associated with a live birth in the current study setting with euploid blastocysts. Morphological evaluation of blastocysts has been widely used for fresh and frozen-warmed ET. Although blastocyst grading systems are the most commonly used method for embryo selection, the relationships between each blastocyst morphology parameter, such as the degree of expansion, ICM score or TE score, and the IVF/ICSI outcome are not well defined. Several studies have reported that TE score has a stronger predictive power than ICM score in the estimation of outcome after blastocyst transfer [49–51], while others have shown that the ICM score is more important [52, 53]. However, some studies have indicated that the clinical outcomes after eSET could be predicted by the degree of blastocoel expansion [54, 55]. A limited number of studies have attempted to correlate conventional parameters of blastocyst evaluation with euploid FBT cycles. In 2014, Capalbo et al. reported that vitrified-warmed euploid embryos of poor or average quality can result in similar ongoing pregnancy rates to those of blastocysts evaluated as having excellent or good morphological quality [56]. This study reported that ICM score and TE score were also not related to the implantation outcomes of euploid embryos. In agreement with this study, we also found that in single euploid FBT, the TE and ICM scores were not related to the live birth outcome.

There is an ongoing debate on the implantation potential of slow-growing embryos. Whether the poorer reproductive potential is due to the pace of embryo development or is a result of asynchronization between the embryo and the endometrium is still poorly understood. The meta-analysis reported in 2010 included 15 controlled studies comprising 2,502 frozen-warmed transfers involving blastocysts that were cryopreserved on either day-5 or day-6 and found no differences in the ongoing pregnancy or live birth rates after the transfer of day-5 frozen blastocysts versus that of day-6 blastocysts with the same morphological quality on the day of cryopreservation [57]. Yang et al. reported that the pregnancy rates were similar between day-5 and day-6 euploid FETs, with similar morphological parameters [58]. In a retrospective cohort follow-up study including 1,347 single autologous FETs, researchers reported that live birth rates were significantly lower with day-6 than with day-5 blastocysts, regardless of embryo quality [59]. On the other hand, Irani et al. analyzed 701 euploid eSETs and determined that, with respect to the day of TE biopsy (day-5 vs day-6), there was a significant difference in the live birth rates of similarly graded euploid blastocysts [60]. The speed of embryo development to the blastocyst stage may reflect not the euploidy status but the metabolic health of a developing embryo. This study confirms that the timing of the blastulation of a euploid embryo influences the live birth rate.

The cause of miscarriage is multifactorial, with genetic, anatomical, infectious, immunological and endocrine causes. In our retrospective study, we included patients with RM, although there have been no randomized trials evaluating the efficacy of PGT-A in RM. An intent-to-treat analysis comparing PGT-A to expectant management reported that the clinical outcomes including live birth and clinical miscarriage rates, were similar between RM patients undergoing PGT-A and those receiving expectant management [61]. However, the role of aneuploidy in RM is less clear. In a retrospective analysis, 428 PGT-A cycles employing FISH were analyzed in cases with RM of unknown etiology and reported that PGT-A should be recommended in cases with miscarriages during infertility treatments, aneuploidy in a previous miscarriage, up to five previous miscarriages or a high incidence of chromosomal abnormalities in spermatozoa [62]. The aneuploidy rates in POC from RM patients have long been evaluated, and there are conflicting results. Some authors reported more frequent aneuploidy [63], while others reported nonsignificantly higher aneuploidy rates observed in sporadic miscarriages, suggesting that additional factors may play a role [64, 65]. In our multivariate analysis, the number of previous miscarriages was an independent factor for the live birth rate, whereby an increasing number of miscarriages decreases the live birth rate. Factors other than aneuploidy, such as immunological factors, may have a detrimental effect on the live birth rate for RM patients.

The mtDNA ratio has been suggested to be a biomarker of embryonic competence and viability. Initial studies reported that an increased mtDNA copy number is related to aneuploidy and a decreased embryo implantation potential [23, 26]. However, there have been other studies contradicting these findings [66, 67]. All of these studies used different methodologies. In this retrospective analysis, mtDNA copy number was found to be nonsignificant in the multivariate analysis. Identification of the best methodologies to measure mtDNA content and factors that lead to the decrease in the mtDNA ratio in blastocyst-stage embryos is also necessary.

FET is an essential part of IVF/ICSI. In a retrospective analysis, the factors that affected the live birth outcome after FET which included cleavage stage embryos, were top-quality embryo characteristics, maternal age, endometrial preparation protocol, number of embryos transferred and BMI [68]. In our study of single euploid FET, BMI, number of previous miscarriages and day of embryo biopsy were found to be independent factors that can affect the live birth rate. Once a euploid embryo is identified, the implantation potential and miscarriage risk are not related to maternal age or blastocyst quality.

This study has limitations. The first is its retrospective design; therefore, a certain risk of bias was inevitable. Second, this study did not observe other clinical outcomes, such as the miscarriage rate. Third, the present study only evaluated the vitrified blastocyst, with a certain blastocoel expansion, after embryo warming. Finally, the study is restricted in a certain ethnic patient population and the data are related to the clinical and lab practices of one unit and the conclusions may reflect the end results of these practices.

In conclusion, BMI, day of TE biopsy and number of miscarriages are the independent variables that affect the live birth outcomes of single euploid FBTs.
